# Metabolomic biomarkers in midtrimester maternal plasma can accurately predict the development of preeclampsia

**DOI:** 10.1038/s41598-020-72852-4

**Published:** 2020-09-30

**Authors:** Seung Mi Lee, Yujin Kang, Eun Mi Lee, Young Mi Jung, Subeen Hong, Soo Jin Park, Chan-Wook Park, Errol R. Norwitz, Do Yup Lee, Joong Shin Park

**Affiliations:** 1 Department of Obstetrics and Gynecology, Seoul National University College of Medicine, Seoul, 03080 Korea; 2 Department of Agricultural Biotechnology, Center for Food and Bioconvergence, Research Institute for Agricultural and Life Sciences, Seoul National University, Seoul, 08826 Korea; 3 Department of Obstetrics and Gynecology, Tufts University School of Medicine, Boston, MA USA

**Keywords:** Biomarkers, Cardiology, Risk factors

## Abstract

Early identification of patients at risk of developing preeclampsia (PE) would allow providers to tailor their prenatal management and adopt preventive strategies, such as low-dose aspirin. Nevertheless, no mid-trimester biomarkers have as yet been proven useful for prediction of PE. This study investigates the ability of metabolomic biomarkers in mid-trimester maternal plasma to predict PE. A case–control study was conducted including 33 pregnant women with mid-trimester maternal plasma (gestational age [GA], 16–24 weeks) who subsequently developed PE and 66 GA-matched controls with normal outcomes (mid-trimester cohort). Plasma samples were comprehensively profiled for primary metabolic and lipidomic signatures based on gas chromatography time-of-flight mass spectrometry (GC-TOF MS) and liquid chromatography Orbitrap mass spectrometry (LC-Orbitrap MS). A potential biomarker panel was computed based on binary logistic regression and evaluated using receiver operating characteristic (ROC) analysis. To evaluate whether this panel can be also used in late pregnancy, a retrospective cohort study was conducted using plasma collected from women who delivered in the late preterm period because of PE (n = 13) or other causes (n = 21) (at-delivery cohort). Metabolomic biomarkers were compared according to the indication for delivery. Performance of the metabolomic panel to identify patients with PE was compared also to a commonly used standard, the plasma soluble fms-like tyrosine kinase-1/placental growth factor (sFlt-1/PlGF) ratio. In the mid-trimester cohort, a total of 329 metabolites were identified and semi-quantified in maternal plasma using GC-TOF MS and LC-Orbitrap-MS. Binary logistic regression analysis proposed a mid-trimester biomarker panel for the prediction of PE with five metabolites (SM C28:1, SM C30:1, LysoPC C19:0, LysoPE C20:0, propane-1,3-diol). This metabolomic model predicted PE better than PlGF (AUC [95% CI]: 0.868 [0.844–0.891] vs 0.604 [0.485–0.723]) and sFlt-1/PlGF ratio. Analysis of plasma from the at-delivery cohort confirmed the ability of this biomarker panel to distinguish PE from non-PE, with comparable discrimination power to that of the sFlt-1/PlGF ratio. In conclusion, an integrative metabolomic biomarker panel in mid-trimester maternal plasma can accurately predict the development of PE and showed good discriminatory power in patients with PE at delivery.

## Introduction

Preeclampsia (PE) is a clinical syndrome specific to human pregnancy and the puerperium that can affect virtually every maternal organ system. It is diagnosed by the presence of hypertension with evidence of end-organ involvement, such as proteinuria, thrombocytopenia, renal insufficiency, pulmonary edema, or cerebral symptoms after 20 weeks’ gestation^1^. Preeclampsia complicates 2–10% of all pregnancies and is a major cause of maternal mortality^2^.

Because of its clinical significance, the prediction and prevention of PE has been the target of intensive investigation for decades. Numerous biological markers have been evaluated in an effort to predict PE, but results have been inconsistent^3–10^. Such biomarkers included those related to placental perfusion/vascular resistance, endocrine dysfunction, renal dysfunction, endothelial dysfunction, and oxidative stress^11^. Overall, these biomarkers have shown poor sensitivity and poor positive-predictive values for the prediction of PE. A test using plasma biomarkers of endothelial dysfunction—specifically the ratio of the anti- and pro-angiogenic factors, soluble fms-like tyrosine kinase-1 (sFlt-1) and placental growth factor (PlGF), respectively—is now commercially available. Although the sFlt-1/PlGF ratio has shown a high degree of accuracy for predicting PE in late pregnancy^12^, the clinical utility of this test in the mid-trimester remains questionable. There are currently no widely accepted biological markers that have been shown to be reliable, reproducible, and cost-effective in the prediction of PE in early pregnancy, which would be critical if a preventative strategy is to be effective.

The metabolome refers to the complete profile of metabolites within the maternal plasma that reflect ongoing biochemical process at a single point in time, including metabolic substrates, intermediates, and final products. Metabolomic analysis of samples collected from study subjects with/without underlying diseases is thus ideally suited for developing diagnostic tests, prognostic tools, and personalized therapeutic interventions^13–15^. Metabolomic studies have been conducted for the purpose of biomarker discovery for PE in a range of biological specimens (including serum, urine, and placenta) and different analytical platforms have been used to measure a number of putative biomarker molecule species including amino acid, fatty acid, organic acid, and acylcarnitine^16,17^, which have been summarized in detail elsewhere^16^. In brief, several studies with maternal blood in late pregnancy reported metabolic biomarkers for discrimination of preeclampsia^18–21^, and other previous studies suggested predictive metabolic biomarkers in early pregnancy with the use of metabolomics tools such as nuclear magnetic resonance spectroscopy (NMR), gas-/ liquid chromatography–mass spectrometry (GC– / LC–MS), with variable sensitivities and specificities (30–100%)^22–29^. Other biologic samples such as urine or placenta in early or late pregnancy has been also explored to examine the underlying pathophysiology of preeclampsia^30–34^.

However, few studies have explored comprehensive phospholipid profiles in maternal blood. Phospholipids (PLs) are a major constituent of cell membranes and function also as second messengers in intracellular signal transduction cascades. The different composition of fatty acyl chains within PL subtypes determines the biophysical traits of the individual cell membrane and influences a range of biological processes^35^. Considering that endothelial injury/dysfunction is a key pathogenic feature of PE^36^, it is biologically plausible that changes in circulating PL profiles might be useful in predicting and/or diagnosing the disease. In addition, abnormal circulating PL profiles have been reported in many diseases, including obesity, hypertension, seizure disorders, and gestational diabetes, all of which are risks factors for PE^37–39^.

In this study, we analyzed comprehensive plasma PL profiles and primary metabolomic signatures from women who did and did not develop PE. We identified unique metabolomic features in women with PE, which was consistent in both early and late gestational ages, and describe a biomarker panel based on binary logistic regression model that showed robust performance in differentiating women with PE from those without.

## Results

### Metabolomic biomarkers in the Mid-trimester Cohort

**Figure 1:**
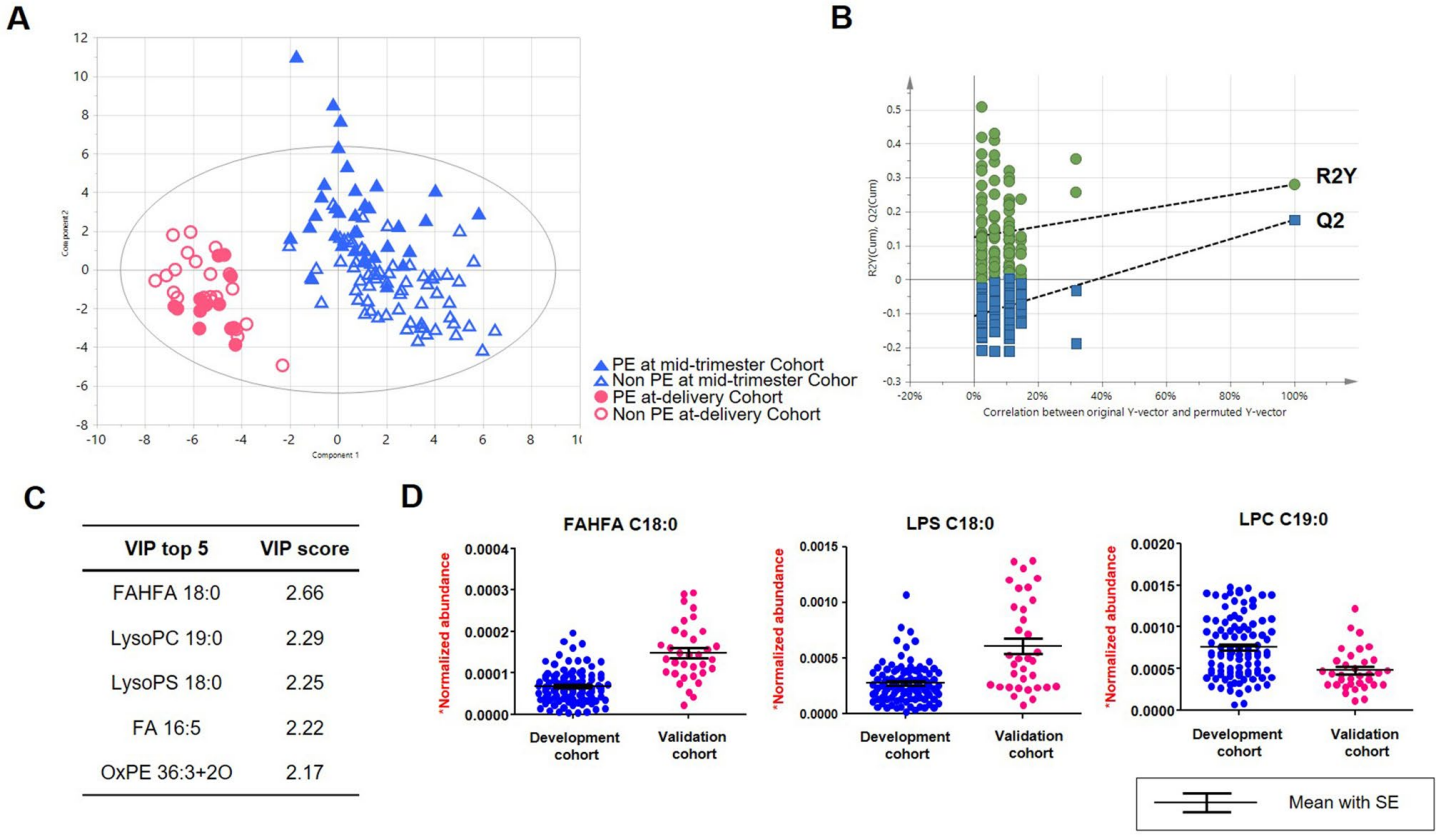
Multivariate statistical modeling of plasma metabolites by partial least squares-discriminant analysis (PLS-DA). (**A**) The score plot shows that the major discriminatory factor is pregnancy stage. T1 and T2 indicates are the vectors, which explain the two largest degree of variation in the model. R2Y (0.425) is cumulative goodness-of-fit. Q2 (0.179) proposes model predictability. (**B**) Random permutation plot (100 times). The vertical axis presents R2 (green points) and Q2 (blue points) values of the model. The horizontal axis shows the correlation coefficient between the original and the permuted Y-variable. (**C**) The top five list of metabolites based on VIP analysis (**D**) Individual measurements as well as Box-Whisker plots (mean ± SEM) are shown of the metabolites that contributed most to the model.

Integrative metabolomic analysis identified and semi-quantified 116 primary metabolites and 213 lipids based on GC-TOF MS and LC-Orbitrap MS, respectively. The profiles were not clearly separated by principal component analysis (PCA) (Figure S1). Supervised multivariate statistics, PLS-DA modeling demonstrated that the primary discriminatory factor was GA at sampling based on the first two latent variables (Fig. 1A,B). Variable importance projection (VIP) analysis determined that the metabolites that contributed most to the model were Fatty Acid Esters of Hydroxy Fatty Acid (FAHFA) C18:0, LysoPC C19:0, and LysoPS C19:0 (Fig. 1C,D). Sixty-one metabolites (18.5%) achieved statistical significance based on one-way ANOVA (adjusted by Benjamini–Hochberg analysis, FDR < 0.05) with post-hoc test (Fisher’s LSD) of which 26 metabolites were significantly different in all 4 comparisons (Table S1). Subsequent PLS-DA showed the PE-unique metabolic profiles in the mid-trimester cohort (R2Y = 0.626 and Q2 = -0.0187) (Figure S2 A,B) and in the at-delivery cohort (R2Y = 0.991 and Q2 = 0.546) (Figure S2 C,D).

We first interrogated the clinical characteristics and the metabolic compositional differences in the mid-trimester cohort. Table 1 shows the clinical characteristics of the mid-trimester cohort. Cases and controls were matched for maternal age and GA at blood sampling. As expected, cases (women who developed PE) had an earlier GA at delivery and lower birthweight that those who did not (controls). Neither circulating sFlt-1 concentrations nor sFlt-1/PlGF ratios were different between the two groups, but PlGF levels were significantly lower in women who subsequently developed PE as compared with those that did not (median 43.1 [range, 3.2–387.8] vs 60.3 [11.3–654.4] pg/mL, respectively; *p* < 0.05).

**Table 1:** Clinical characteristics of the study population in the mid-trimester cohort.

	Controls (did not develop preeclampsia) (n = 66)	Cases (developed preeclampsia) (n = 33)	*p *Value
Maternal age (years)	36 (27–43)	35 (26–44)	NS
Nulliparity	39 (59%)	20 (61%)	NS
BMI before pregnancy	20.7 (16.4–32.8) (n = 51)	22.4 (17.6–40.2) (n = 31)	< 0.01
BMI at blood sampling	22.4 (16.7–36.5)	23.8 (18.2–38.1)	NS
Gestational age at blood sampling (weeks)	17.4 (16.0–23.4)	17.6 (16.0–22.6)	NS
Gestational age at delivery (weeks)	39.3 (25.6–42.3)	37.4 (25.3–41.3)	< 0.001
Birth weight (g)	3275 (760–4260)	2670 (450–4700)	< 0.001
Small for gestational age	4 (6%)	6 (19%)	NS
Sex (male)	34 (53%)	16 (49%)	NS
Cesarean delivery	26 (41%)	16 (49%)	NS
Diabetes during pregnancy	1 (2%)	0 (0%)	NS
**Pro- and anti-angiogenic factors**			
sFlt-1 (pg/mL)	887.4 (177.3–3446.2)	952.2 (273.7–1957.1)	NS
PlGF (pg/mL)	60.3 (11.3–654.4)	43.1 (3.2–387.8)	< 0.05
sFlt-1/PlGF	15.1 (0.8–239.2)	18.0 (1.5–577.8)	NS

All values are given as median (range) or number (%).

NS, not significant; BMI, body mass index; PlGF, placental growth factor; sFlt-1, soluble fms-like tyrosine kinase-1.

The compositional changes in the metabolic profiles were interrogated based on Student’s *t*-test in which 6 primary metabolites (1.8%) and 17 lipid molecules (5.2%) were present in plasma at significantly different levels in PE cases compared to healthy controls (*p* < 0.05) (listed in Table 2). The primary metabolites included glycolysis intermediates (3-phosphoglycerate), nitrogenous compounds (xanthine, glutamate), and palmitoleic acid, all of which were elevated in women who developed PE. Lyxose and propane-1,3-diol were present at significantly lower levels in women who developed PE. Metabolite enrichment analysis suggested that arachidonic acid metabolism, malate-aspartate shuttle, β-alanine metabolism were significantly altered (*p *value < 0.05) (Figure S3).

**Table 2:** Metabolites that were significantly different in the preeclampsia group in the mid-trimester cohort.

	Fold change	*p *Value	FDR
**Lipid molecules**			
LysoPC C19:0	0.72	0.002	0.000
LysoPC C22:1	0.53	0.010	0.000
LysoPE C16:1	1.73	0.018	0.783
LysoPE C17:0	1.30	0.013	0.783
LysoPE C20:0	1.46	0.044	0.783
OxPC C38:4 + 1O	0.76	0.048	0.336
OxPI C38:4 + 1O	0.73	0.044	0.277
PC C32:1	1.37	0.007	0.783
PC C32:2	1.33	0.009	0.783
PE C23:1e	0.65	0.030	0.231
PE C24:1e	0.46	0.031	0.231
PE C34:3e	0.81	0.031	0.277
PI C36:2	0.72	0.014	0.231
PI C38:3	11.23	0.041	0.783
SM C28:1	0.66	0.011	0.000
SM C30:1	1.53	0.020	0.783
SM C34:1	2.22	0.044	0.783
**Primary metabolites**			
3-Phosphoglycerate	1.37	0.037	0.783
Glutamate	1.29	0.041	0.783
Lyxose	0.74	0.024	0.231
Palmitoleic acid	1.50	0.046	0.844
Propane-1,3-diol	0.91	0.028	0.231
Xanthine	1.43	0.044	0.783

* Statistical significance is presented by *p *value and false discovery rate (FDR) against control.

From the lipidomic profiles, phosphatidylinositol (PI) C38:3 was increased the most (11.2 fold-change). Levels of lyso-phosphatidylethanolamine (LysoPEs, C16:1, C17:0, and C20:0) were also measured at higher levels in the PE group, whereas levels of phosphatidylethanolamine (PE) C23:1 and C24:1, and C34:3 were significantly reduced. Similarly, phosphatidylcholine (PC) and LysoPC showed contrasting patterns, with lower contractions of LysoPC C19:0 and C22:1 and higher concentrations of PC C32:1 and C32:2 in the PE group. Other lipid molecules that were differentially regulated included oxidized PLs (OxPC and OxPI) and sphingomyelins (SMs). The identities of the oxidized PLs were validated by predicted spectra based on CFM-ID^40^.

**Figure 2:**
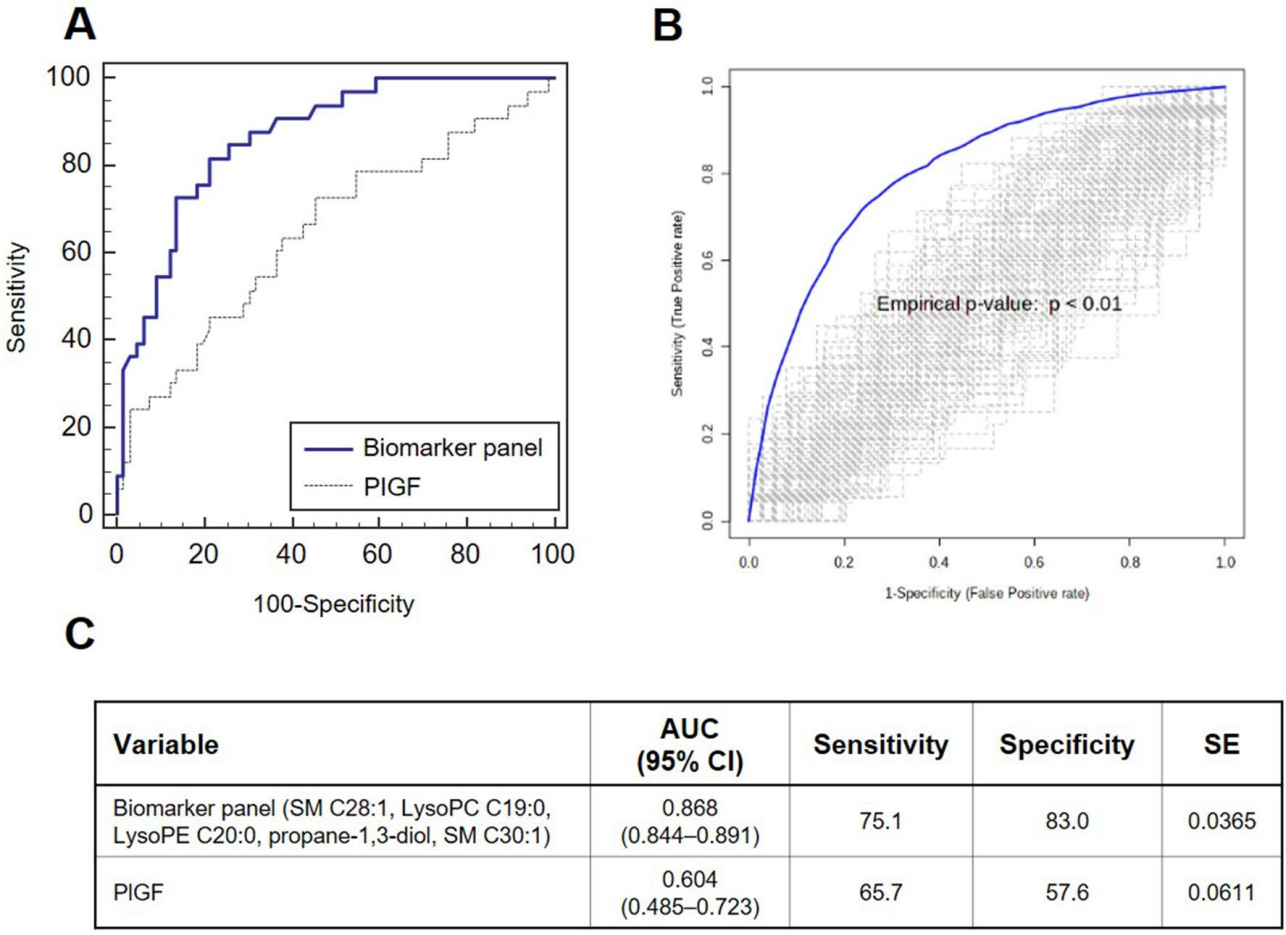
Performance comparison of the metabolic biomarker panel and PlGF for discriminating preeclampsia from healthy controls in the mid-trimester cohort (**A**) Receiver operating characteristic (ROC) curve analysis. The biomarker panel of 5 discriminatory circulating metabolites were tested against PlGF levels in their ability to predict the development of PE. (**B**) Validation based on permutation test with 100 time-random sampling (**C**) Summarized features of ROC curve analysis of two models.

Next, we examined if blood metabolites can discriminate PE from healthy control in mid-trimester cohort. Initially, we tested the metabolite recomposite based on the PLS-DA model; however, the panel did not show good performance (AUC: 0.793) (Figure S4). Alternatively, we explored linearly-recomposited metabolite panel derived based on binary logistic regression analysis. The multivariate logistic model suggested five metabolites (SM C28:1, SM C30:1, LysoPC C19:0, LysoPE C20:0, propane-1,3-diol) and presented a risk of developing PE = -0.626 + (-1.015*SM C28:1) + (1.546*SM C30:1) + (-1.043*LysoPC C19:0) + (0.994*LysoPE C20:0) + (-0.713*propane-1,3-diol) (Figure S5 A). ROC curve analysis confirmed good performance of the metabolic signature (AUC: 0.868, 95% confidence interval [CI]: 0.844–0.891, Sensitivity: 75.1, Specificity: 83.0) (Fig. 2). We also compared the area under the ROC curve between the combined metabolic prediction model and PlGF levels for the ability to predict PE. The combined metabolic model consisting of five metabolites (SM C28:1, SM C30:1, LysoPC C19:0, LysoPE C20:0, propane-1,3-diol) predicted the risk of PE better than PlGF concentrations (AUC [95% CI]: 0.868 [0.844–0.891] vs 0.604 [0.485–0.723]).

### Metabolic biomarkers in the at-delivery cohort

We next performed a comprehensive metabolic profile analysis from maternal plasma collected at delivery. Table 3 shows the clinical characteristics of the at-delivery cohort. Of these, 13 women (38%) were delivered because of PE. With the exception of birth weight, clinical characteristics were not different between women who were delivered for PE or for other indications. Circulating sFlt-1 concentrations and sFlt-1/PlGF ratios were significantly higher and PlGF concentrations significantly lower in the group delivered for PE (Table 3).

**Table 3:** Clinical Characteristics of the Study Population in the At-delivery Cohort.

	Group 1	Group 2	*p* Value
	Delivered for reasons other than preeclampsia (n = 21)	Delivered for preeclampsia (n = 13)	
Maternal age (years)	32 (28–42)	31 (22–40)	NS
Nulliparity	5 (24%)	6 (46%)	NS
BMI before pregnancy	20.9 (15.2–29.6) (n = 13)	22.4 (18.4–25.6) (n = 8)	NS
BMI at delivery	25.9 (20.3–33.3) (n = 17)	27.7 (24.8–31.0) (n = 10)	NS
Gestational age at delivery (weeks)	35.4 (34.3–36.9)	35.6 (34.1–36.9)	NS
Birth weight (g)	2660 (1710–3440)	2160 (1560–3240)	< 0.01
Small for gestational age	1 (5%)	2 (15%)	NS
Sex (male)	11 (52%)	6 (46%)	NS
Cesarean delivery	17 (81%)	13 (100%)	NS
Diabetes during pregnancy	2 (10%)	0 (0%)	NS
**Pro- and anti-angiogenic factors**			
sFlt-1 (pg/mL)	1325.0 (199.5–9884.9)	2791.6 (723.8–7887.2)	< 0.05
PlGF (pg/mL)	31.1 (3.0–256.3)	3.1 (1.6–40.7)	< 0.001
sFlt-1/PlGF	32.8 (7.1–3278.0)	856.2 (59.6–3243.8)	< 0.001

All values are given as median (range) or number (%).

NS, not significant; BMI, body mass index; PlGF, placental growth factor; sFlt-1, soluble fms-like tyrosine kinase-1.

A comparative analysis of the metabolomic profiles revealed 17 lipids (5.2%) and 7 primary metabolites (2.1%) that were significantly different in women delivered with PE versus no PE (Student’s *t*-test, listed in Table 4). Of these, the major lipid classes included sphingomyelins (C30:1, C32:1, C33:2, C34:2, and C38:1), that were present at higher levels in the PE versus non-PE group, as well as FAHFA (C18:0), PCs, PE, LysoPC, and LysoPEs, most of which were also significantly higher in the PE group. Similarly, all identified primary metabolites were upregulated in the PE group. Remarkably, the disaccharide, isomaltose, showed a more than two-fold increase in PE. Other primary metabolites of note included 2-deoxytetronic acid, 3,6-anhydro-d-galactose, sugar alcohols (erythritol, xylitol, and myo-inositol), and thymine (Table 4). Isomaltose and erythritol that were originated from dietary resource, have been suggested for the association with disease biomarker, particularly in diabetes^41,42^.

**Table 4:** Metabolites That Were Significantly Different in the Preeclampsia Group in the At-delivery Cohort.

Metabolites list	Fold change	*p* Value	FDR
**Lipid molecules**			
FAHFA C18:0	1.41	0.028	0.331
LysoPC C18:2	0.44	0.007	0.580
LysoPE C15:0	0.28	0.043	0.589
LysoPE C18:2	0.31	0.037	0.589
LysoPE C20:4	0.63	0.049	0.589
OxPC C38:4 + 1O(1Cyc)	1.72	0.003	0.000
OxPE C38:3 + 1O	1.86	0.001	0.000
OxPI C38:4 + 1O	1.52	0.028	0.331
PC C36:5e	1.65	0.042	0.331
PC C38:5	1.89	0.009	0.098
PC C40:7	1.82	0.008	0.098
PE C36:4	2.73	0.010	0.098
SM C30:1	2.37	0.001	0.000
SM C32:1	1.80	0.012	0.098
SM C33:2	2.63	0.033	0.331
SM C34:2	1.43	0.049	0.331
SM C38:1	2.18	0.049	0.331
**Primary metabolites**			
2-Deoxytetronic acid	1.55	0.035	0.193
3,6-Anhydro-d-galactose	1.28	0.029	0.193
Erythritol	1.31	0.043	0.315
Isomaltose	2.37	0.028	0.193
Myo-inositol	1.39	0.014	0.193
Thymine	1.24	0.048	0.315
Xylitol	1.21	0.032	0.193

*Statistical significance is presented by p-value and false discovery rate (FDR) against control.

**Figure 3:**
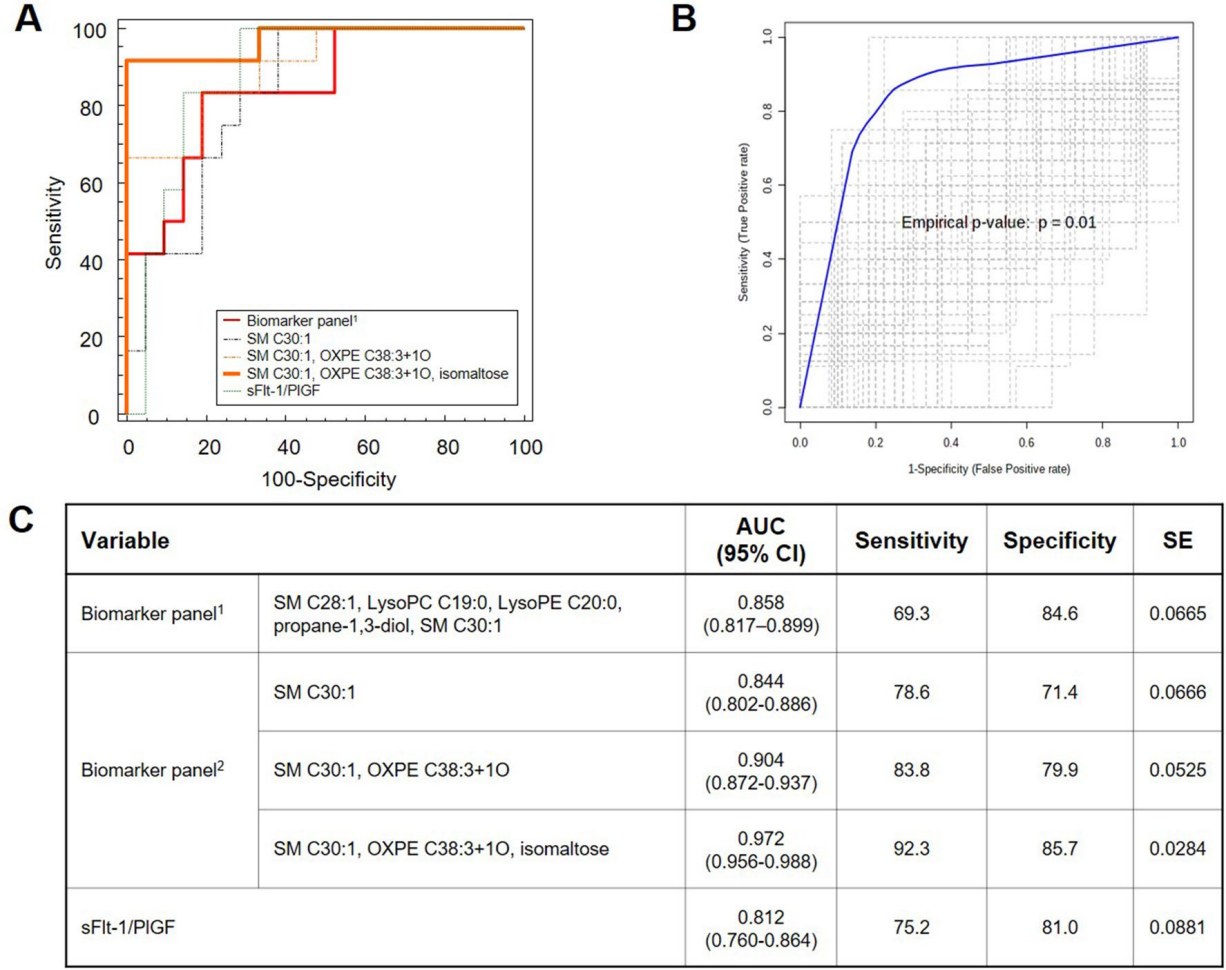
Performance comparison of the metabolic biomarker panels and sFlt-1/PlGF ratio for discriminating preeclampsia from non-preeclampsia in the at-delivery cohort. (**A**) Receiver operating characteristic (ROC) curve analysis. The same metabolic biomarker derived from the mid-trimester cohort study is re-formulated for at-delivery cohort using binary logistic regression analysis (biomarker panel^1^). New biomarker panel was generated based metabolite set determined from at-delivery cohort study (biomarker panel^2^). (**B**) Validation based on permutation test with 100 time-random sampling (**C**) Summarized features of ROC curve analysis of the tested models.

Next, we set out to confirm whether the panel of 5 metabolites identified in the mid-trimester cohort could discriminate between cases with and without PE in the at-delivery cohort. The same panel of 5 compounds (SM C28:1, SM C30:1, LysoPC C19:0, LysoPE C20:0, and propane-1,3-diol) showed good discriminatory performance, with similar AUC to that of the sFlt-1/PlGF ratio (AUC [95% CI]: 0.858 [0.817–0.899] vs AUC [95% CI]: 0.812 [0.760–0.864]) (Fig. 3, Figure S5 B).

Then, we further explored if there is a combination of metabolomic markers that could more accurately distinguish between PE and non-PE in the at-delivery cohort. All metabolites that were significantly different in the PE group were analyzed (Table 4) and prioritized using binary logistic regression. The analysis suggested three consecutive models, which consisted of SM C30:1 alone, SM C30:1 + oxidized PE (OxPE) C38:3, and SM C30:1 + oxidized PE (OxPE) C38:3 + isomaltose. The calculated AUC (95% CI) values were as follows: 0.844 (0.802–0.886), 0.904 (0.872–0.937), and 0.972 (0.956–0.988), respectively. The model with the three metabolites (SM C30:1 + oxidized PE (OxPE) C38:3 + isomaltose) showed the marginal difference (p = 0.08) compared to the sFlt-1/PlGF ratio (0.812 [0.760–0.864]) (Fig. 3). Ten-fold cross validation was performed for each model and provided in Figure S6.

## Discussion

In the current study, we comprehensively characterized the metabolome in mid-trimester maternal plasma and identified a panel composed of 5 metabolites that can accurately predict the subsequent development of PE. We then tested this same biomarker signature in a second cohort of women delivered in the late preterm period, and were able to accurately discriminate women who were delivered with PE from those delivered with other indications. In addition, we first reported the significant differences in metabolic phenotype according to gestational age at sampling (mid-trimester and at-delivery), suggesting that these biomarkers may be used for both prediction and diagnosis along with pregnancy period. Particularly, lipid molecules, FAHFA C18:0 and LysoPC C19:0 were highly abundant at the time of delivery.

In mid-trimester population, we obtained predictive model for PE with 5 plasma metabolites based on binary logistic regression following the variable prioritization based on Pattern Searching algorithm^43^. The regression model presented good predictive performance in the mid-trimester period (16–24 weeks) for the subsequent development of PE. The biomarker signature included SM C28:1, SM C30:1, LysoPC C19:0, LysoPE C20:0, and propane-1,3-diol. Aberrant levels of SMs, which serve as important structural components of cell membranes and as bioactive signal transduction molecules in various cellular response^44^, have been measured in fetal (umbilical artery) blood^45^, placentas^46^, and maternal blood^46^ collected from pregnancies complicated by PE.

A number of intervention strategies have been proposed to prevent PE including dietary manipulation, exercise, cardiovascular drugs, and antioxidants, but none has been found to be effective. Low-dose aspirin (LDA), which acts in part by inhibiting platelet thromboxane A_2_ biosynthesis, is the one intervention that has been shown to reduce the development of PE in some high-risk patients^47–49^. As such, recent guidelines support the use of LDA starting from mid-pregnancy period to prevent PE in high-risk populations, including women with a history of PE in a prior pregnancy, and women with multiple gestations, chronic hypertension, pregestational diabetes, renal disease, or autoimmune diseases^1,50^. However, the use of LDA in low-risk populations is not currently recommended even though substantial number of the women without these risk factors can develop PE during pregnancy^51^.

For the reasons outlined above, the early risk assessment of PE development is highly desirable for low-risk population. Indeed, the application of complex algorithms (e.g. maternal historical and demographic risk factors, blood pressure, uterine artery pulsatility index on Doppler velocimetry, and maternal serum biomarkers such as pregnancy-associated plasma protein A [PAPP-A] and PlGF) to identify women at risk for preeclampsia and use of LDA might also lead to a reduction of PE development^52^. The current study suggests that a new test using maternal plasma biomarkers alone can more accurately identify women at high-risk of PE development and would allow for more timely and appropriate utilization of preventive medications for low-risk pregnant women (those without a history of prior PE and those with no underlying medical conditions). Most guidelines recommend the LDA therapy starting at the late first or early second trimester of pregnancy^1,50^. Therefore, early and accurate identification of women with high risk for PE is critical to allow for the timely adoption of LDA prophylaxis. In the current study, we developed simple binary logistic regression model composed of 5 plasma metabolites, which accurately predicted the development of PE superior to PIGF. The simpler model using only SMs (SM C28:1 and SM C30:1) also showed good performance for the PE prediction. Moreover, our highly quantitative and comprehensive approach suggested the plausible association between the metabolic features and potential patho-mechanism. For instance, the increases in circulating SMs has been described in a lipidomic analysis of placental microvesicles from abnormal pregnancies and appears to correlate with an increase in inflammation and oxidative stress^53^. The additional examination of plasma metabolites from the at-delivery cohort implied an underlying aberrant redox regulation given the significant increase in oxidized PLs (OxPC, OxPI, and OxPE) in the PE group. A recent report suggested that this may be due to the enhanced release of the anti-angiogenic factor, endoglin, by circulating exosome in response to exposure to high levels of SMs^53^. Indeed, in our model, the combination of SM C30:1 and OxPE C38:3 was best able to discriminate between patients with and without PE at delivery. The pathophysiologic mechanism regarding these biomarkers needs to be further investigated for exploring novel therapeutic targets.

The appropriate development of the prediction model would be completed through the validation of the developed model in independent study population, but we could not split the study population into the development and validation set because of small number of cases. Instead we validated the model in the same study population and tested if the developed model can be also used in the identification of preeclampsia patient at delivery. Further studies with large number of cases with separate development and validation population should be performed. For the clinical application, the biomarker also needs to be validated in other cohorts including the group in the first trimester. As guidelines recommend the start of LDA ideally in the late first trimester^49,50^, the first trimester biomarkers would be clinically more applicable. In addition, the comparative evaluation of prediction accuracy between the metabolic biomarkers and clinical parameters, or the combination of both needs to be evaluated in future studies.

In addition, we evaluated the usefulness of metabolomic panel in at-delivery cohort. The identical biomarker composite developed from the mid-trimester cohort also showed good discrimination between PE and non-PE in the late preterm study population at delivery (at-delivery cohort). Furthermore, the metabolic panel derived from at-delivery cohort showed the outperformance in the discriminant power. For at-delivery cohort, we evaluated the maternal plasma at delivery from patients who delivered in the late preterm period (34–0/7—36–6/7 weeks) as the following reasons: (1) Guidelines recommend that women with severe preeclampsia should be delivered at or after 34 weeks of gestation^54^. Therefore most patients with severe preeclampsia are intended to deliver at late preterm period; (2) In addition, to compare the pathophysiologic changes in biomaterials of preeclampsia with those of control pregnancy, proper comparison should be performed between pregnant women with preeclampsia and those who delivered at term without any complications, but this comparison can be biased by the difference of gestational age at delivery. To adjust gestational age at delivery, most studies included pregnant women who delivered at the similar gestational age (preterm) after spontaneous preterm labor or rupture of membranes. However, the result from this comparison can be difficult in interpretation, because the changes in biomaterials could be originate from the parturition mechanism in preterm labor/rupture of membranes, as well as from the pathophysiologic mechanism of preeclampsia. To overcome this problem, we included as controls not only women who delivered preterm after spontaneous parturition but also those who delivered preterm because of maternal medical indication in late preterm period.

In conclusion, we demonstrated that a combination of 5 blood metabolites in mid-trimester maternal plasma accurately predicted the development of PE. This unique metabolomic panel was further validated by its accurate predictability that discriminated the patients delivered in the late preterm period for PE from those delivered for other indications.

## Methods

### Study design

#### (1) Mid-trimester cohort

The population for this case–control study consisted of 33 healthy pregnant women whose plasma was taken in the mid-trimester period (gestational age [GA] range 16–24 weeks, median 17.4 weeks) and who subsequently developed PE [cases]. Control subjects included 66 pregnant women with mid-trimester plasma samples who did not develop PE and were matched for maternal age and GA at maternal plasma sampling (ratio 1:2) [controls]. Patients with major fetal malformations and multifetal pregnancies were excluded. The maternal plasma metabolite and PL profiles were compared between cases and controls. PE was defined as the development of hypertension with evidences of end-organ involvement such as proteinuria, low platelet count, renal or liver involvement, cerebral symptoms, or pulmonary edema^1^.

#### (2) At-delivery cohort

After characterization of the metabolomic biomarker panel, we evaluated the discriminatory power of this panel of biomarkers to identify women with PE at the time of delivery. The latter study population consisted of singleton pregnant women who delivered in the late preterm period (34–0/7 to 36–6/7 weeks), had maternal plasma collected within 3 days of delivery, and had no evidence of chorioamionitis. Study subjects were divided into 2 groups according to the indication for preterm birth (i.e., PE or other causes such as spontaneous preterm labor and maternal and/or fetal indication). The plasma metabolomic biomarker measurements were compared between subjects who did and did not have PE at delivery.

### Metabolomic profiling of maternal plasma

Maternal plasma samples were centrifuged at 2,000 rpm for 10 min and the supernatant stored at -70 °C until assayed. Samples were comprehensively analyzed for primary metabolomic and lipidomic signatures using gas chromatography time-of-flight mass spectrometry (GC-TOF MS) and liquid chromatography Orbitrap mass spectrometry (LC-Orbitrap MS) [See Appendix S1 for further details of methodology]^55–57^. In addition, all analytical procedures, including extraction, reconstitution, and MS analysis were performed in random order to minimize potential systematic errors. The study was approved by the Institutional Review Board of Seoul National University Hospital. All patients provided written informed consent for sample collection and the use of biologic materials for research purposes. All methods were performed in accordance with the relevant guidelines and regulations including Declaration of Helsinki.

### Measurement of sFlt-1 and PlGF

For comparison, sFlt-1 and PlGF levels were measured in all samples using a highly sensitive multiplex array in accordance with the instruction of the manufacturer (Meso Scale Discovery (MSD) V-PLEX Angiogenesis Panel 1 Human kit, MSD, USA).

### Statistical analysis

For clinical information, continuous data were analyzed using the Mann–Whitney U test and categorical data using the Fisher’s exact test or Chi-square test, as appropriate. Statistical analyses were conducted using the IBM SPSS version 20 (IBM Corp., Armonk, NY, USA).

The integrative metabolomic profiles were pre-processed for data normalization using the MSTUS methodology^58^ implemented in NOREVA (https://idrb.zju.edu.cn/noreva/)^59^. MSTUS (MS Total Useful Signal) is a method of summing and adjusting the total intensities of identified metabolites across all samples). Student’s *t*-test was performed for univariate statistics using EXCEL (Microsoft Office 2010). One-way ANOVA was conducted with post-hoc testing (Fisher’s LSD) as indicated. For adjusting multiple comparisons, the false discovery rate (FDR) was computed (adjusted by Benjamini-Hochberg). Partial least squares-discriminant analysis (PLS-DA) and permutation test were carried out for multivariate statistics using SIMCA 15 (Umetrics AB, Umea, Sweden). Binary logistic regression was performed with forward or enter selection, as appropriate, for predictive model construction using IMB SPSS Statistics for Windows, version 25.0 (IBM Corp.) following auto-scaling procedure. Receiver operating characteristic (ROC) analysis, tenfold cross validation, permutation test, and enrichment analysis were performed using Metaboanalyst 4.0 (https://www.metaboanalyst.ca/).

## Supplementary information

Supplementary file1

Supplementary file2

Supplementary file3

## Data Availability

The datasets analyzed during the current study are not publicly available, but are available from the corresponding author on reasonable request.
