# Supplementary material for: Metabolomic biomarkers in midtrimester maternal plasma can accurately predict the development of preeclampsia
Source: Sci Rep. 2020 Sep 30;10:16142. doi: 10.1038/s41598-020-72852-4 (PMC7527521; doi:10.1038/s41598-020-72852-4)
Supplement: Supplementary file 1 — Supplementary file1 [file 41598_2020_72852_MOESM1_ESM.doc]

**Metabolomic Biomarkers In Midtrimester Maternal Plasma Can**

**Accurately Predict The Development of Preeclampsia**

Seung Mi Lee, MD, PhD,^1^ Yujin Kang, MS,^2^ Eun Mi Lee, MS,^3^ Young Mi Jung, MD,^1^ Subeen Hong, MD,^1^

Soo Jin Park,^2^ MS, Chan-Wook Park, MD, PhD,^1^ Errol R. Norwitz, MD, PhD,^4^

Do Yup Lee, PhD,^3*^ Joong Shin Park, MD, PhD^1*^

^1^Department of Obstetrics and Gynecology, Seoul National University College of Medicine, Seoul, Korea

^2^Department of Bio and Fermentation Convergence Technology, BK21 PLUS Program, Kookmin University, Seoul, Korea

^3^Department of Agricultural Biotechnology, Center for Food and Bioconvergence, Research Institute for Agricultural and Life Sciences, Seoul National University, Seoul, Korea

^4^Department of Obstetrics and Gynecology, Tufts University School of Medicine, Boston, MA, U.S.A

**Figure S1. Score scatter plot based on principal component analysis**

**
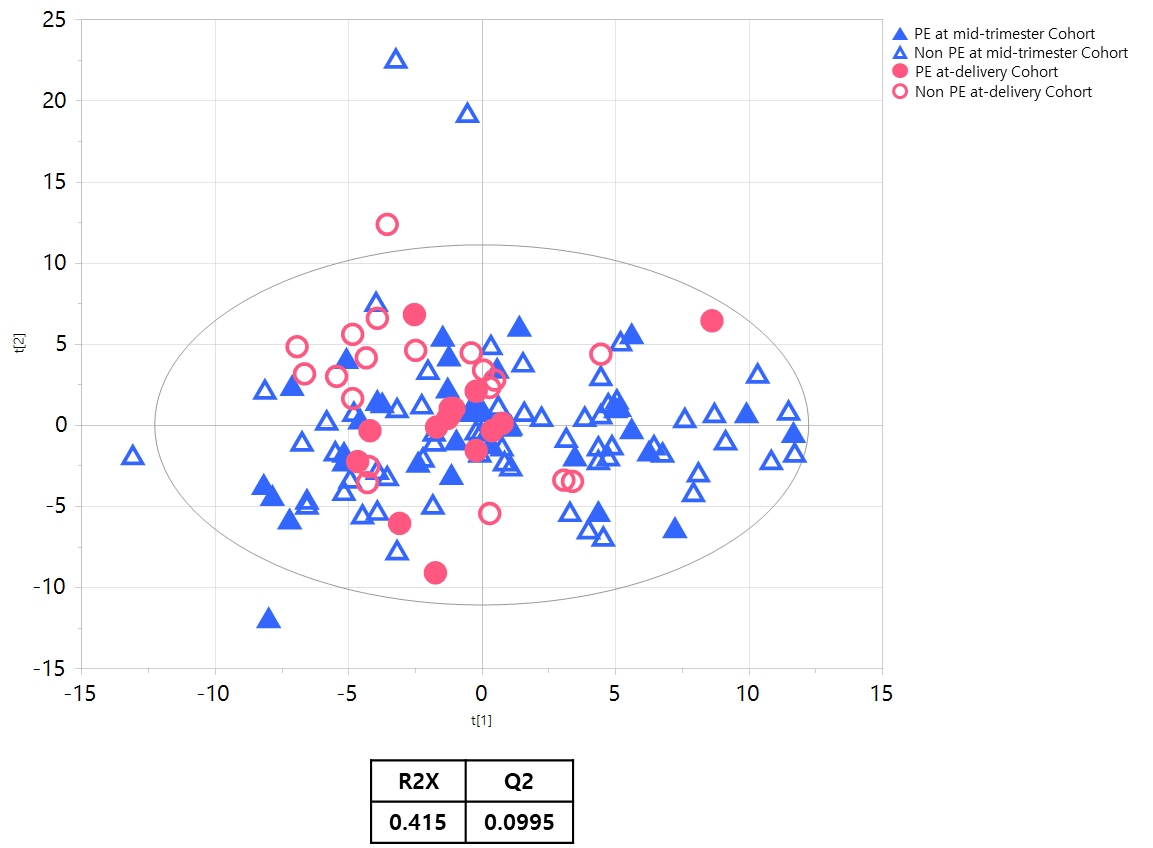
**

**Figure S2. Partial least squares-discriminant analysis for the metabolic profiles of (A, B) mid-trimester cohort and (C, D) at-delivery cohort.**

**
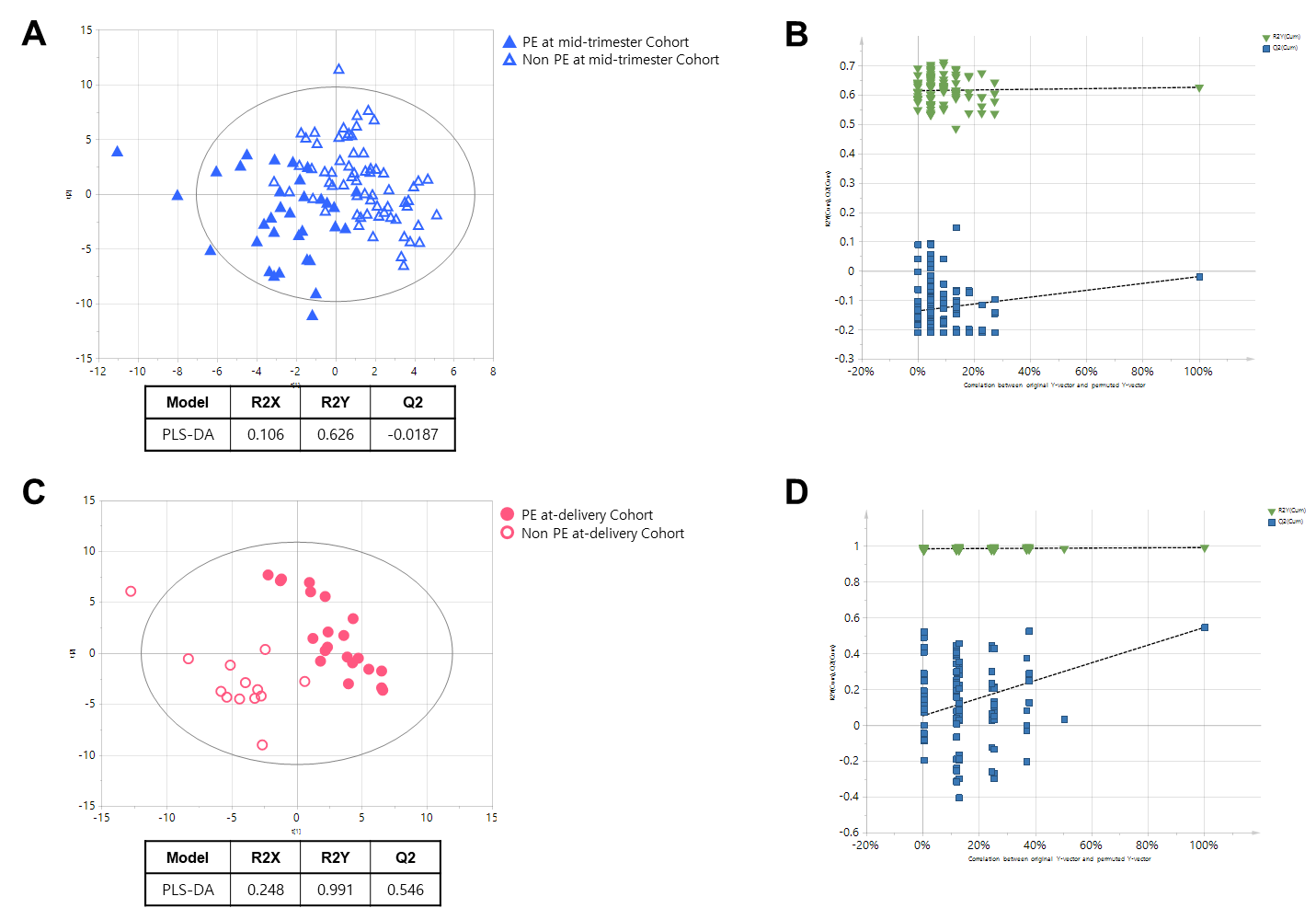
**

**Figure S3. Metabolite enrichment analysis of PE at mid-trimester cohort based on the module in MetaboAnalyst. * indicates significant difference in PE compared to healthy controls**

**
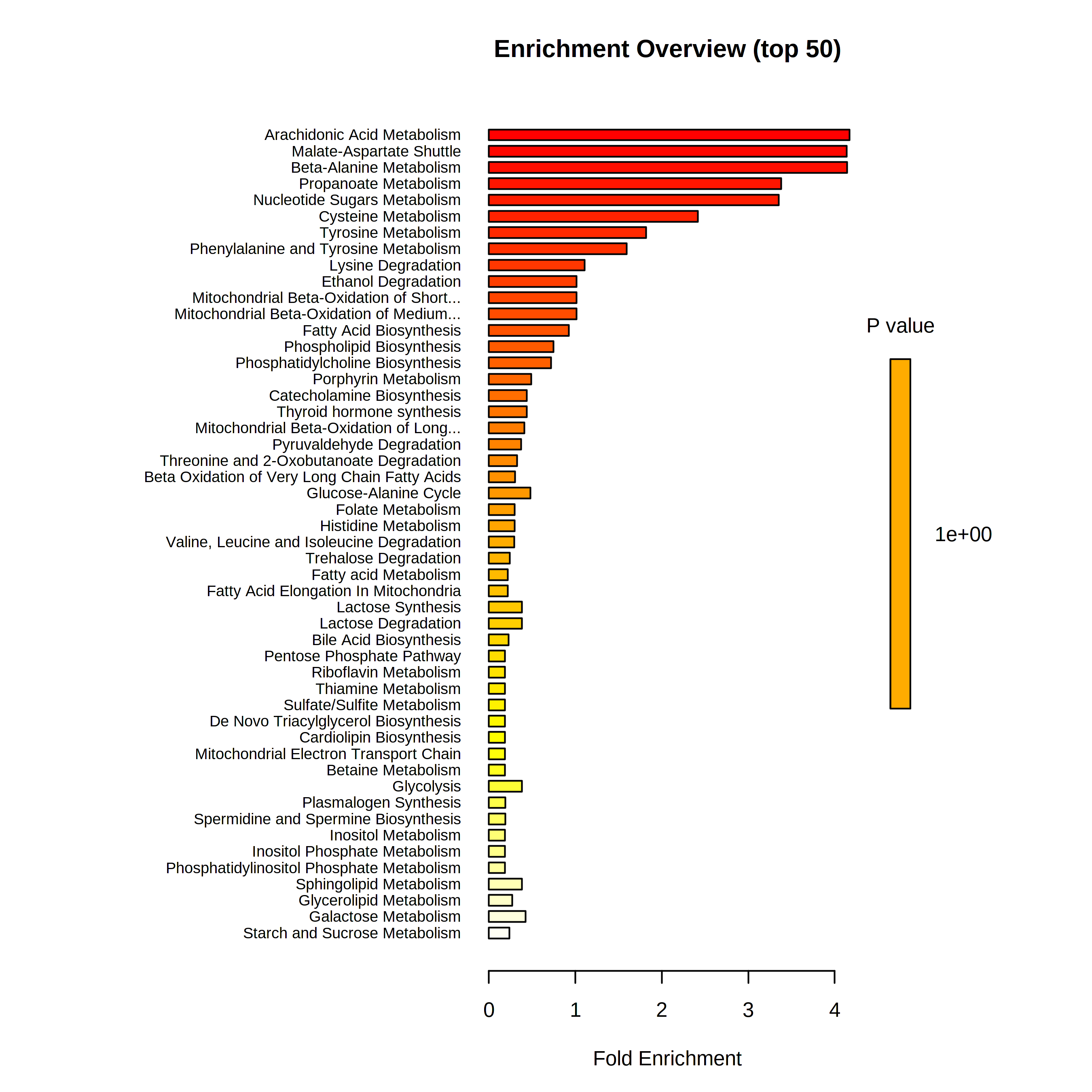
**

**Figure S4. ROC curve analysis of the metabolic panel composed of top metabolites prioritized by variable importance in projection (VIP) analysis in mid-trimester cohort.**

**
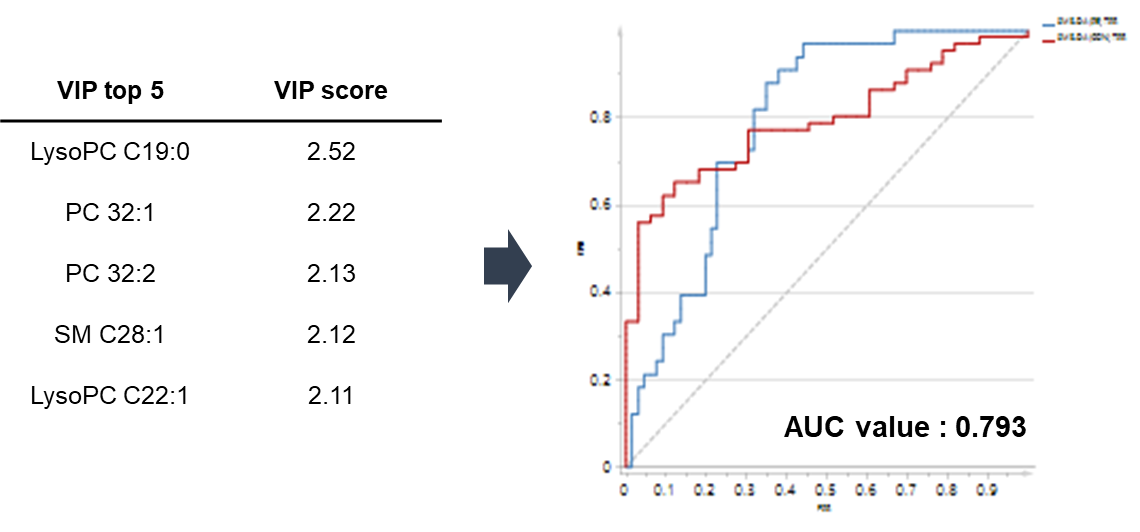
**

**Figure S5. Summary of each feature of logistic regression model. (A) Biomarker for mid-trimester cohort (B) Biomarker for at-delivery cohort derived from mid-trimester (C) Biomarker for at-deliver cohort derived from at-delivery cohort**

**
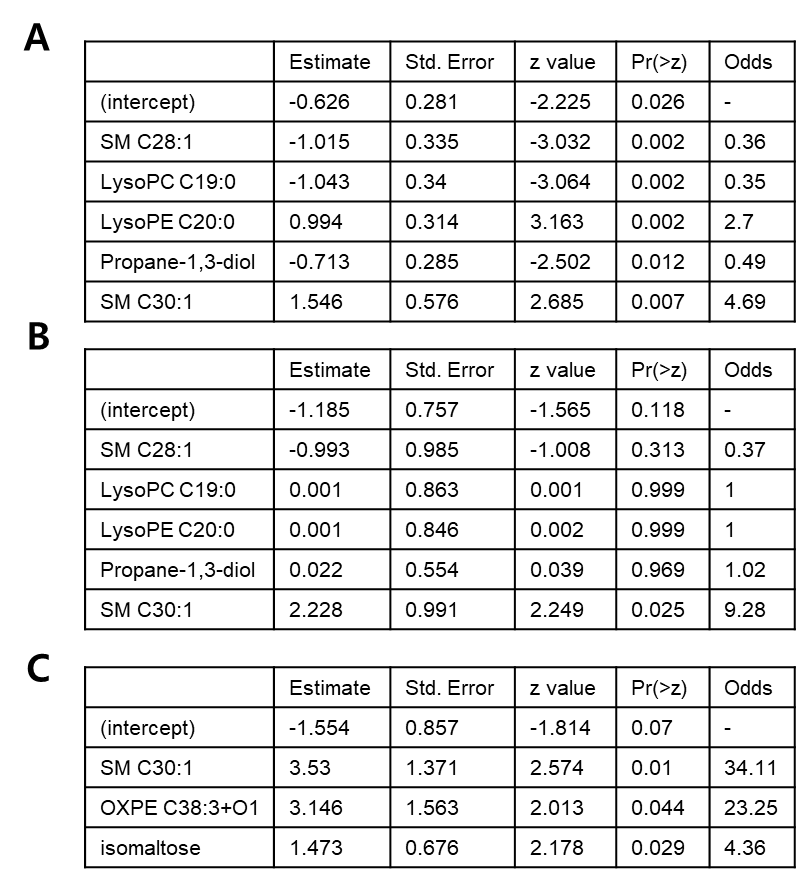
**

**Figure S6. K(10)-fold cross-validation of receiver operating characteristic curve analysis. (A) Biomarker for mid-trimester cohort (B) Biomarker for at-delivery cohort derived from mid-trimester (C) Biomarker for at-deliver cohort derived from at-delivery cohort (D) AUCs with sensitivity and specificity**

**
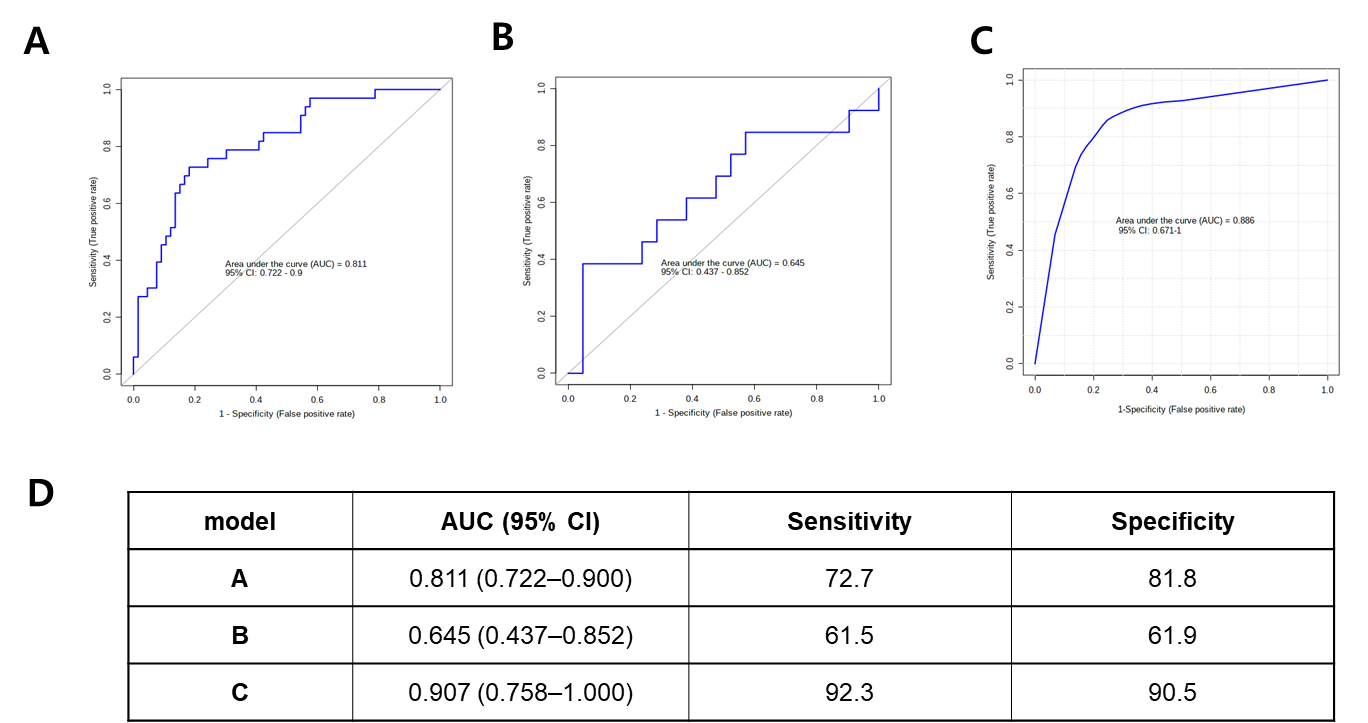
**

**Figure S7. Score scatter plot (T1) computed based on principal component analysis of pooling samples for quality control purpose**

**
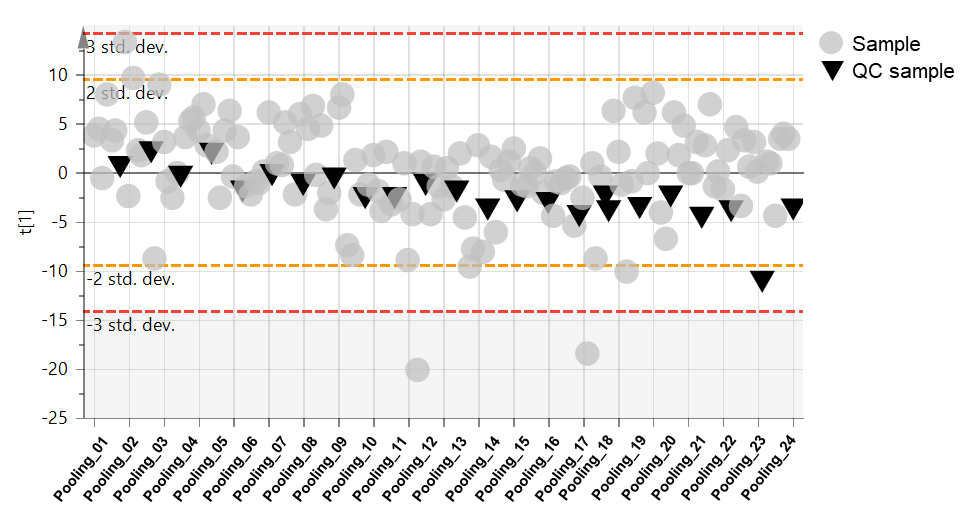
**
