# Supplementary material for: Metabolomic biomarkers in midtrimester maternal plasma can accurately predict the development of preeclampsia
Source: Sci Rep. 2020 Sep 30;10:16142. doi: 10.1038/s41598-020-72852-4 (PMC7527521; doi:10.1038/s41598-020-72852-4)
Supplement: Supplementary file 2 — Supplementary file2 [file 41598_2020_72852_MOESM2_ESM.doc]

**Metabolomic Biomarkers In Midtrimester Maternal Plasma Can**

**Accurately Predict The Development of Preeclampsia**

Seung Mi Lee, MD, PhD,^1^ Yujin Kang, MS,^2^ Eun Mi Lee, MS,^3^ Young Mi Jung, MD,^1^ Subeen Hong, MD,^1^ Soo Jin Park,^2^ MS, Chan-Wook Park, MD, PhD,^1^ Errol R. Norwitz, MD, PhD,^4^ Do Yup Lee, PhD,^3*^ Joong Shin Park, MD, PhD^1*^

^1^Department of Obstetrics and Gynecology, Seoul National University College of Medicine, Seoul, Korea

^2^Department of Bio and Fermentation Convergence Technology, BK21 PLUS Program, Kookmin University, Seoul, Korea

^3^Department of Agricultural Biotechnology, Center for Food and Bioconvergence, Research Institute for Agricultural and Life Sciences, Seoul National University, Seoul, Korea

^4^Department of Obstetrics and Gynecology, Tufts University School of Medicine, Boston, MA, U.S.A

**Appendix S1**

**GC-TOF MS analysis** Plasma samples (50 µL) were thawed on ice and mixed with 750 µL of methanol:isopropanol:water, 3:3:2). The mixtures were sonicated for 10 min and centrifuged for 5 min (13,200 rpm at 4°C). Supernatants (700 µL) was aliquoted into a new 1.5-mL tube and dried to complete in a speed vacuum concentrator (SCANVAC, Korea). The dried extracts were derivatized with 5 µL of 40 mg/mL methoxyamine hydrochloride (Sigma-Aldrich, St. Louis, MO, USA) in pyridine (Thermo, USA) (90 min at 800 rpm at 30°C). The second derivatization was done by mixing 45 µL of N-methyl-N-trimethylsilyltrifluoroacetamide (MSTFA + 1% TMCS; Thermo, USA) . Fatty acid methyl esters (2 µL, FAMEs) was added to the derivatization mixture as retention time index. The derivatives of 0.5ul were injected using an Agilent 7890B (Agilent Technologies, Wilmington, DE, USA). The metabolites were separated by a RTX-5Sil MS column (Restek, Gellefonte, PA, USA) installed in the Agilent 7890B gas chromatograph. Mass spectrometry analysis was conducted on a Leco Pegasus HT time of flight mass spectrometer controlled by Chroma TOF software 4.50 version (LECO, St. Joseph, MI, USA). Data pre-preprocessing was done using ChromaTOF software upon data acquisition, and post-processing was done based on *Binbase* algorithm including chromatogram validation, primary RI detection and calculation, and validation of quant mass. A total of 116 primary metabolites were identified. A mixture of 33 pure standard compounds was analyzed every 8 samples for quality control purpose.

**LC-Orbitrap MS analysis for phospholipid profiling** For lipid profiling, Folch method was applied with minor modification. Briefly, plasma samples (50 µL) were mixed with 225 µL of cold MeOH and then vortexed for 10 sec. Chloroform (450 µL) was added and then incubated for 60 min followed by phase separation with 187.5 µL of water. A lower phase was aliquoted (350 µL) and transferred to new 1.5-ml tubes. The aliquots were completely dried in a speed vacuum concentrator (SCANVAC, Korea) and reconstituted with 50 µL of acetonitrile (70%) for MS analysis**.**

The lipid reconstituent was separated by binary solvent system consisting of solvent A (water with 10 mM ammonium formate and 0.2% formic acid) and solvent B (acetonitrile with 0.2% formic acid). The gradient was as follows: equilibration in 10% solvent B for 1 min, 10–75% solvent B gradient until 7.5 min, 75–95% solvent B gradient until 7.6 min, 95% solvent B until 10.4 min, 95-10% solvent B gradient until 10.5min, and re-equilibration in 10% solvent B until 16 min. MS analysis was performed using Q-Exactive Plus Orbitrap (ionization mode: negative, Scan parameter: full scan/ddMS2, scan range: 70-1,000 m/z, resolution: 70,000, automatic gain control: 1e6, maximum injection time: 100 ms, HESI-II voltage: 3500v, S-lens radio frequency level: 50 v, capillary temperature: 300°C). MS/MS analysis was conducted in data-dependent manner (HCD, 30 eV). Raw data (.raw format) was transformed to Analysis Base File (ABF) format by Reifycs Abf Converter (http://www.reifycs.com/AbfConverter/index.html). Data process (compound identification and semi-quantification) was done by the MS-DIAL software and Lipid Blast library (MS1 tolerance : 0.005Da, MS2 tolerance : 0.01Da, similarity score: 75%). A total of 213 lipids were identified. Pooling samples were analyzed every 6 samples for QC purpose (Figure S7).
