# Supplementary material for: Metabolomic biomarkers in midtrimester maternal plasma can accurately predict the development of preeclampsia
Source: Sci Rep. 2020 Sep 30;10:16142. doi: 10.1038/s41598-020-72852-4 (PMC7527521; doi:10.1038/s41598-020-72852-4)
Supplement: Supplementary file 3 — Supplementary file3 [file 41598_2020_72852_MOESM3_ESM.doc]

**Metabolomic Biomarkers In Midtrimester Maternal Plasma Can**

**Accurately Predict The Development of Preeclampsia**

Seung Mi Lee, MD, PhD,^1^ Yujin Kang, MS,^2^ Eun Mi Lee, MS,^3^ Young Mi Jung, MD,^1^ Subeen Hong, MD,^1^ Soo Jin Park,^2^ MS, Chan-Wook Park, MD, PhD,^1^ Errol R. Norwitz, MD, PhD,^4^ Do Yup Lee, PhD,^3*^ Joong Shin Park, MD, PhD^1*^

^1^Department of Obstetrics and Gynecology, Seoul National University College of Medicine, Seoul, Korea

^2^Department of Bio and Fermentation Convergence Technology, BK21 PLUS Program, Kookmin University, Seoul, Korea

^3^Department of Agricultural Biotechnology, Center for Food and Bioconvergence, Research Institute for Agricultural and Life Sciences, Seoul National University, Seoul, Korea

^4^Department of Obstetrics and Gynecology, Tufts University School of Medicine, Boston, MA, U.S.A

**Table S1.** Metabolites that were differentially regulated among all 4 groups based on one-way ANOVA.*

| Metabolites list | f-value | p-value | -log 10 | FDR | Post-hoc test (Fisher's LSD) |
| --- | --- | --- | --- | --- | --- |
| Lipid molecules |  |  |  |  |  |
| FA C16:5 | 14.210 | 4.66E-08 | 7.331 | 5.11E-06 | OAM_HC - AM_DC, OAM_PE - AM_DC, OAM_HC - AM_PE, OAM_PE - AM_PE |
| FA C20:4 | 9.140 | 1.58E-05 | 4.802 | 3.46E-04 | OAM_HC - AM_DC, OAM_PE - AM_DC, OAM_HC - AM_PE, OAM_PE - AM_PE |
| FA C22:7 | 11.194 | 1.41E-06 | 5.852 | 5.14E-05 | OAM_HC - AM_DC, OAM_PE - AM_DC, OAM_HC - AM_PE, OAM_PE - AM_PE |
| FAHFA C18:0 (1) | 28.318 | 3.90E-14 | 13.408 | 1.28E-11 | AM_PE - AM_DC,  AM_DC - OAM_HC,  AM_DC - OAM_PE,  AM_PE - OAM_HC,  AM_PE - OAM_PE |
| FAHFA C18:0 (2) | 4.519 | 4.77E-03 | 2.322 | 3.08E-02 | AM_DC - OAM_PE,  AM_PE - OAM_HC,  AM_PE - OAM_PE |
| LysoPC C16:2 | 7.608 | 1.01E-04 | 3.998 | 1.50E-03 | OAM_HC - AM_DC, OAM_HC - AM_PE, OAM_HC - OAM_PE |
| LysoPC C18:2 | 10.817 | 2.18E-06 | 5.662 | 6.52E-05 | AM_DC - AM_PE,  OAM_HC - AM_DC, OAM_PE - AM_DC, OAM_HC - AM_PE, OAM_PE - AM_PE |
| LysoPC C19:0 | 10.019 | 5.55E-06 | 5.255 | 1.52E-04 | OAM_HC - AM_DC, OAM_HC - AM_PE, OAM_HC - OAM_PE |
| LysoPC C20:0 | 11.362 | 1.16E-06 | 5.936 | 4.77E-05 | OAM_HC - AM_DC, OAM_PE - AM_DC, OAM_HC - AM_PE, OAM_PE - AM_PE, OAM_HC - OAM_PE |
| LysoPC C20:4 | 4.133 | 7.79E-03 | 2.108 | 4.58E-02 | AM_DC - OAM_HC,  AM_DC - OAM_PE,  AM_PE - OAM_HC |
| LysoPC C20:5 | 4.221 | 6.97E-03 | 2.157 | 4.24E-02 | OAM_HC - AM_PE, OAM_PE - AM_PE |
| LysoPC C22:1 | 4.730 | 3.65E-03 | 2.438 | 2.55E-02 | OAM_HC - AM_DC, OAM_HC - AM_PE, OAM_HC - OAM_PE |
| LysoPC C22:2 | 8.963 | 1.95E-05 | 4.711 | 4.00E-04 | OAM_HC - AM_DC, OAM_PE - AM_DC, OAM_HC - AM_PE, OAM_HC - OAM_PE |
| LysoPC C22:5 | 5.727 | 1.04E-03 | 2.985 | 1.06E-02 | OAM_HC - AM_DC, OAM_PE - AM_DC, OAM_HC - AM_PE, OAM_PE - AM_PE |
| LysoPC C24:1 | 6.994 | 2.14E-04 | 3.670 | 2.81E-03 | OAM_HC - AM_DC, OAM_PE - AM_DC, OAM_HC - AM_PE, OAM_PE - AM_PE |
| LysoPE C16:0 | 9.818 | 7.04E-06 | 5.153 | 1.78E-04 | AM_DC - OAM_HC,  AM_DC - OAM_PE,  AM_PE - OAM_HC |
| LysoPE C17:0 | 10.974 | 1.82E-06 | 5.741 | 5.98E-05 | AM_DC - OAM_HC,  AM_DC - OAM_PE,  AM_PE - OAM_HC |
| LysoPE C18:1 | 4.855 | 3.11E-03 | 2.507 | 2.23E-02 | AM_DC - AM_PE,  AM_DC - OAM_HC,  AM_DC - OAM_PE |
| LysoPE C20:1 | 6.276 | 5.22E-04 | 3.283 | 6.36E-03 | OAM_HC - AM_DC, OAM_PE - AM_DC, OAM_HC - AM_PE |
| LysoPE C20:4 | 7.874 | 7.27E-05 | 4.138 | 1.20E-03 | OAM_HC - AM_DC, OAM_PE - AM_DC, OAM_HC - AM_PE, OAM_PE - AM_PE |
| LysoPE C20:5 | 4.604 | 4.28E-03 | 2.369 | 2.86E-02 | OAM_HC - AM_DC, OAM_HC - AM_PE, OAM_PE - AM_PE |
| LysoPE C22:1 | 5.335 | 1.70E-03 | 2.770 | 1.43E-02 | OAM_HC - AM_DC, OAM_PE - AM_DC, OAM_HC - AM_PE, OAM_PE - AM_PE |
| LysoPI C20:4 | 4.333 | 6.04E-03 | 2.219 | 3.75E-02 | OAM_HC - AM_DC, OAM_PE - AM_DC, OAM_HC - AM_PE, OAM_PE - AM_PE |
| LysoPS C18:0 | 14.922 | 2.14E-08 | 7.670 | 3.52E-02 | AM_DC - OAM_HC,  AM_DC - OAM_PE,  AM_PE - OAM_HC,  AM_PE - OAM_PE |
| OxPC C34:1+1O(1Cyc) | 4.619 | 4.20E-03 | 2.377 | 2.86E-02 | AM_DC - OAM_PE,  AM_PE - OAM_HC,  AM_PE - OAM_PE |
| OxPC 38:4+1O(1Cyc) | 4.078 | 8.35E-03 | 2.078 | 4.74E-02 | OAM_HC - AM_DC, OAM_HC - AM_PE, OAM_HC - OAM_PE |
| OxPE C34:2+1O | 7.637 | 9.71E-05 | 4.013 | 1.50E-03 | AM_PE - AM_DC,  AM_PE - OAM_HC,  AM_PE - OAM_PE |
| OxPE C36:3+1O | 7.398 | 1.30E-04 | 3.886 | 1.78E-03 | AM_PE - AM_DC,  AM_DC - OAM_HC,  AM_PE - OAM_HC,  AM_PE - OAM_PE |
| OxPE C36:3+2O | 12.226 | 4.31E-07 | 6.366 | 2.83E-05 | AM_DC - OAM_HC,  AM_DC - OAM_PE,  AM_PE - OAM_HC,  AM_PE - OAM_PE |
| OxPE C38:4+1O (1) | 5.289 | 1.80E-03 | 2.745 | 1.48E-02 | OAM_HC - AM_DC, OAM_PE - AM_DC |
| OxPE C38:4+1O (2) | 4.150 | 7.62E-03 | 2.118 | 4.56E-02 | OAM_HC - AM_DC, OAM_PE - AM_DC, OAM_PE - AM_PE |
| OxPE 38:4+3O(2Cyc) | 6.178 | 5.89E-04 | 3.230 | 6.92E-03 | AM_DC - OAM_HC,  AM_DC - OAM_PE,  AM_PE - OAM_HC,  AM_PE - OAM_PE |
| OxPE C38:5+1O | 5.846 | 8.93E-04 | 3.049 | 9.79E-03 | OAM_HC - AM_DC, OAM_PE - AM_DC, OAM_HC - AM_PE, OAM_PE - AM_PE |
| OxPE C40:6+1O | 9.195 | 1.48E-05 | 4.831 | 3.46E-04 | OAM_HC - AM_DC, OAM_PE - AM_DC, OAM_HC - AM_PE, OAM_PE - AM_PE |
| OxPI C38:4+1O | 5.104 | 2.27E-03 | 2.644 | 1.74E-02 | OAM_HC - AM_DC, OAM_HC - OAM_PE |
| PC C32:1 | 5.065 | 2.39E-03 | 2.622 | 1.78E-02 | OAM_PE - AM_DC, OAM_PE - AM_PE,  OAM_PE - OAM_HC |
| PC C32:2 | 4.047 | 8.70E-03 | 2.061 | 4.80E-02 | OAM_PE - AM_DC, OAM_PE - AM_PE,  OAM_PE - OAM_HC |
| PC C34:3e | 4.591 | 4.35E-03 | 2.362 | 2.86E-02 | OAM_HC - AM_DC, OAM_PE - AM_DC, OAM_HC - AM_PE |
| PC C35:3 | 5.735 | 1.03E-03 | 2.989 | 1.06E-02 | OAM_HC - AM_DC, OAM_PE - AM_DC, OAM_HC - AM_PE, OAM_PE - AM_PE |
| PC C35:4 | 5.957 | 7.77E-04 | 3.110 | 8.81E-03 | OAM_HC - AM_DC, OAM_PE - AM_DC, OAM_HC - AM_PE, OAM_PE - AM_PE |
| PC C36:4e | 5.247 | 1.90E-03 | 2.722 | 1.52E-02 | OAM_HC - AM_DC, OAM_HC - AM_PE |
| PC C36:5e | 5.597 | 1.22E-03 | 2.914 | 1.13E-02 | OAM_HC - AM_DC, OAM_PE - AM_DC |
| PC C38:5 | 7.473 | 1.19E-04 | 3.926 | 1.70E-03 | OAM_HC - AM_DC, OAM_PE - AM_DC, OAM_HC - AM_PE |
| PC C38:5e | 8.438 | 3.66E-05 | 4.436 | 6.70E-04 | OAM_HC - AM_DC, OAM_PE - AM_DC, OAM_HC - AM_PE |
| PC C38:6 | 4.042 | 8.75E-03 | 2.058 | 4.80E-02 | OAM_HC - AM_DC, OAM_HC - AM_PE |
| PC C40:5 | 6.504 | 3.93E-04 | 3.406 | 4.97E-03 | OAM_HC - AM_DC, OAM_PE - AM_DC, OAM_HC - AM_PE |
| PC C40:6 | 5.183 | 2.05E-03 | 2.687 | 1.61E-02 | OAM_HC - AM_DC, OAM_PE - AM_DC, OAM_HC - AM_PE |
| PC C40:7 | 5.674 | 1.11E-03 | 2.956 | 1.10E-02 | OAM_HC - AM_DC, OAM_PE - AM_DC, OAM_HC - AM_PE |
| PC C40:7e | 7.983 | 6.37E-05 | 4.196 | 1.10E-03 | OAM_HC - AM_DC, OAM_PE - AM_DC, OAM_HC - AM_PE, OAM_PE - AM_PE |
| PE C21:0 (1) | 12.292 | 3.99E-07 | 6.399 | 2.83E-05 | AM_DC - AM_PE,  AM_DC - OAM_HC,  AM_DC - OAM_PE,  AM_PE - OAM_PE |
| PE C21:0 (2) | 4.366 | 5.79E-03 | 2.237 | 3.67E-02 | AM_PE - OAM_HC,  AM_PE - OAM_PE |
| PE C23:1 | 11.854 | 6.58E-07 | 6.182 | 3.61E-05 | AM_DC - OAM_HC,  AM_DC - OAM_PE,  AM_PE - OAM_HC,  AM_PE - OAM_PE |
| PE C24:0 | 4.009 | 9.12E-03 | 2.040 | 4.92E-02 | AM_DC - OAM_HC,  AM_PE - OAM_HC,  AM_PE - OAM_PE |
| PE C34:1 | 4.897 | 2.95E-03 | 2.530 | 2.16E-02 | AM_DC - AM_PE,  AM_DC - OAM_HC,  AM_DC - OAM_PE |
| PE C343e | 4.111 | 8.01E-03 | 2.096 | 4.62E-02 | OAM_HC - AM_DC, OAM_HC - OAM_PE |
| PE C36:3e | 5.526 | 1.33E-03 | 2.875 | 1.19E-02 | OAM_HC - AM_DC, OAM_PE - AM_DC, OAM_HC - AM_PE, OAM_PE - AM_PE |
| PI C36:3 | 5.617 | 1.19E-03 | 2.925 | 1.13E-02 | OAM_HC - AM_DC, OAM_HC - AM_PE, OAM_PE - AM_PE |
| SM C28:1 | 8.877 | 2.16E-05 | 4.665 | 4.18E-04 | OAM_HC - AM_DC, OAM_HC - AM_PE, OAM_HC - OAM_PE |
| SM C30:1 | 5.451 | 1.47E-03 | 2.834 | 1.27E-02 | AM_PE - AM_DC,  OAM_PE - AM_DC,  AM_PE - OAM_HC, OAM_PE - OAM_HC |
| SM C30:2 | 11.704 | 7.82E-07 | 6.107 | 3.67E-05 | OAM_HC - AM_DC, OAM_PE - AM_DC, OAM_HC - AM_PE, OAM_PE - AM_PE, OAM_HC - OAM_PE |
| Primary metabolites |  |  |  |  |  |
| Isomaltose | 5.584 | 1.24E-03 | 2.907 | 1.13E-02 | AM_PE - AM_DC,  AM_PE - OAM_HC,  AM_PE - OAM_PE |

* The 4 groups include the cases and controls in both the Development Cohort and the Validation Cohort.

FA, free fatty acid; FAHFA, fatty acid esters of hydroxyl fatty acid; LysoPC, lysophosphatidylcholine; LysoPE, lysophosphatidylethanolamine; LysoPI, lysophosphatidylinositol; LysoPS, lysophosphatidylserine; OxPC, oxidized phosphatidylcholine; OxPE, oxidized phosphatidylethanolamine; OxPI, oxidized phosphatidylinositol; PC, phosphatidylcholine; PE, phosphatidylethanolamine; PI, phosphatidylinositol; SM, sphingomyelin
